# Adenosine A_2A_ Receptor Antagonists Do Not Disrupt Rodent Prepulse Inhibition: An Improved Side Effect Profile in the Treatment of Parkinson's Disease

**DOI:** 10.1155/2012/591094

**Published:** 2011-12-04

**Authors:** Carina J. Bleickardt, Abigail L. LaShomb, Carrie E. Merkel, Robert A. Hodgson

**Affiliations:** Department of In Vivo Pharmacology, Neuroscience, Merck Research Laboratories, 2015 Galloping Hill Road, K-15-1600, Kenilworth, NJ 07033, USA

## Abstract

Parkinson's disease (PD) is characterized by loss of dopaminergic neurons in the substantia nigra. Current treatments for PD focus on dopaminergic therapies, including L-dopa and dopamine receptor agonists. However, these treatments induce neuropsychiatric side effects. Psychosis, characterized by delusions and hallucinations, is one of the most serious such side effects. Adenosine A_2A_ receptor antagonism is a nondopaminergic treatment for PD with clinical and preclinical efficacy. The present studies assessed A_2A_ antagonists SCH 412348 and istradefylline in rodent prepulse inhibition (PPI), a model of psychosis. Dopamine receptor agonists pramipexole (0.3–3 mg/kg), pergolide (0.3–3 mg/kg), and apomorphine (0.3–3 mg/kg) significantly disrupted PPI; ropinirole (1–30 mg/kg) had no effect; L-dopa (100–300 mg/kg) disrupted rat but not mouse PPI. SCH 412348 (0.3–3 mg/kg) did not disrupt rodent PPI; istradefylline (0.1–1 mg/kg) marginally disrupted mouse but not rat PPI. These results suggest that A_2A_ antagonists, unlike dopamine agonists, have an improved neuropsychiatric side effect profile.

## 1. Introduction

Parkinson's disease (PD) is characterized by a progressive loss of dopaminergic neurons in the substantia nigra region of the basal ganglia, which results in movement-related symptoms. Current treatment for PD includes dopamine replacement therapy in the form of L-dopa, a precursor to dopamine (DA) in its synthesis pathway that has been the gold standard of care for decades. More recently, DA receptor agonists, such as pramipexole, pergolide, and ropinirole, have become more commonly prescribed for the treatment of PD.

PD is primarily associated with motor symptoms, but nonmotor neuropsychiatric symptoms have garnered recent attention as serious complications that negatively impact quality of life [1, 2]. Psychosis, mostly in the form of visual hallucinations and sometimes paranoid delusions, is a troubling neuropsychiatric symptom in PD patients. Treatment with dopaminergic medication is a risk factor for developing psychosis. Up to 40% of PD patients treated with dopaminergic agents experience psychotic symptoms, of which the most common manifestations are visual hallucinations [3, 4], whereas less than 10% of untreated PD patients experience psychotic symptoms [5]. Among the antiparkinsonian medications, studies have shown that DA receptor agonists are more likely to induce psychoses than L-dopa [5–7]. First-line treatment for psychosis in PD is typically dose reduction of dopaminergic agents. Second-line treatment is administration of atypical antipsychotics, particularly clozapine and quetiapine [8]. However, these drugs carry the risk of worsening the motor symptoms of PD either by counteracting the dopaminergic treatment effects or inducing extrapyramidal side effects [9]. Better treatment options for PD without the associated psychosis liability would be extremely beneficial.

Given the clinical link between dopaminergic therapies and psychoses, preclinical models of psychosis that are translatable to humans are necessary to predict the psychosis risk of novel PD medications. Prepulse inhibition (PPI) of startle is a preclinical model of sensory gating that we used in the current studies to evaluate this risk for A_2A_ receptor antagonists. Typically, PPI deficits are associated with neuropsychiatric disorders such as schizophrenia. DA receptor agonists disrupt PPI in rats and humans [10–12], which demonstrates the cross-species reliability of the PPI model. These findings also provide evidence that PPI disruptions can be used to predict neuropsychiatric side effects of PD medications.

Adenosine A_2A_ receptor antagonism has recently emerged as a potential novel nondopaminergic treatment for PD. A_2A_ receptors are abundant in the GABAergic neurons of the indirect pathway of the basal ganglia [13]. The location of these receptors suggests that they are potent neuromodulators and may regulate excitatory input to the striatum, which is an important target for PD treatment due to its involvement in the control of voluntary movements [14]. A_2A_ receptor antagonism has proven beneficial in clinical trials. In a recent phase II clinical trial, the A_2A_ antagonist preladenant was found to decrease off time and motor fluctuations in patients with PD receiving L-dopa [15].

A_2A_ receptor antagonists have also demonstrated efficacy in animal models of PD. The A_2A_ receptor antagonist istradefylline (KW-6002) increased locomotor activity in MPTP-treated mice and decreased mouse catalepsy induced by haloperidol or reserpine [16]. Of particular interest to the present studies is SCH 412348, which is a novel and potent A_2A_ antagonist that displays high selectivity (>1000-fold) over all other adenosine receptor subtypes (*K*_*i*_ = 0.6 nM) [17]. SCH 412348 (0.1–1.0 mg/kg) has been shown to potentiate L-dopa-induced rotations in 6-OHDA-lesioned rats and reverse rat haloperidol-induced catalepsy, two rodent models predictive of antiparkinsonian efficacy [17].

The purpose of the current research was to evaluate any potential psychosis liability of A_2A_ antagonists. SCH 412348 and istradefylline were assessed in both rat and mouse PPI and compared to current dopamine-based PD therapies (pramipexole, pergolide, ropinirole, L-dopa, and apomorphine). Doses tested in PPI were based on efficacy in rat haloperidol-induced catalepsy.

## 2. Materials and Methods

### 2.1. Animals

Male CD rats weighing 180–220 g and 250–450 g were used in catalepsy and PPI studies, respectively. Male C57BL/6 mice (20–25 g) were used in mouse PPI studies. Animals were purchased from Charles River Laboratories (Kingston, NY, USA). Animals were group-housed with food and water available ad libitum. Studies were conducted during the light phase of a 12 h light/dark cycle under standard laboratory conditions (constant temperature and humidity). Animal care and testing procedures were conducted in conformity with the Merck Institutional Animal Care and Use Committee, and in compliance with the “Guide for the Care and Use of Laboratory Animals” (National Research Council, 1996) and the Animal Welfare Act.

### 2.2. Drugs

Haloperidol, pergolide mesylate, ropinirole hydrochloride, L-dopa, benserazide, and apomorphine were obtained from Sigma-Aldrich (St. Louis, Mo, USA). Pramipexole*·*2HCl was purchased from Tecoland Corporation (Edison, NJ, USA). For catalepsy studies, haloperidol was prepared with distilled water and brought to a pH of 5-6 with 0.1 N HCl and 0.1 M NaOH. A dose of 1 mg/kg was administered SC 30 min prior to catalepsy testing. SCH 412348 ([7-[2-[4-2,4-difluorophenyl]-1-piperazinyl]ethyl]-2-(2-furanyl)-7H-pyrazolo[4,3-e][1,2, 4]triazolo[1,5-c]pyrimidin-5-amine) and istradefylline [(E)-8-(3,4-dimethoxystyryl)-1,3-diethyl-7-methyl-3,7-dihydro-1H-purine-2,6-dione] were synthesized by the Department of Chemical Research at Merck Research Laboratories. SCH 412348 was prepared in 0.4% methylcellulose and administered orally 60 min prior to behavioral testing. Istradefylline was dissolved in 5% Tween 80 in saline and administered orally 60 min prior to behavioral testing. Pramipexole was dissolved in saline and injected sc. 30 min prior to behavioral testing. Pergolide was prepared in saline and dosed ip. 10 min prior to testing. Ropinirole was dissolved in saline and injected ip. 60 min prior to testing. L-dopa was prepared in saline and administered ip. 60 min prior to catalepsy or PPI testing. Twenty min prior to L-dopa, benserazide dissolved in saline was injected ip. (2 : 1 ratio of L-dopa to benserazide) to prevent peripheral decarboxylation of L-dopa. Apomorphine solution in 0.1% ascorbic acid was administered sc. 5 min prior to PPI testing. In rats, dose volume for oral administration was 5 mL/kg, while dose volume for both sc. and ip. administration was 1 mL/kg. Dose volume for all routes of administration in mice was 10 mL/kg.

### 2.3. Haloperidol-Induced Catalepsy Procedure

The catalepsy procedure followed that described by Hodgson et al., in 2009 [17]. Catalepsy was measured using an angled wire mesh screen (60° angle, 59 cm (W) × 24 cm (D) × 56.0 cm (H); mesh 5 mm^2^). The duration of catalepsy was scored by an experimenter using a hand-held timer. Rats were first injected with haloperidol to induce catalepsy. Thirty minutes later, each rat was placed on the wire mesh screen with its head facing upward and forelimbs and hindlimbs extended. To prescreen the rats to ensure they were responsive to haloperidol, they were given two trials to demonstrate catalepsy (operationally defined as remaining still without lifting a paw from the wire mesh) for 120 sec to meet study inclusion threshold. Haloperidol was not injected a second time for the second trial. Rats that met the criterion (roughly 85% of the rats tested) on at least one of the two trials were injected with the drug of interest and tested for catalepsy after the appropriate pretreatment time. The latency to move a paw was the dependent measure in the catalepsy studies, with all trials truncated at 120 sec. Studies were conducted using a between-subjects design.

### 2.4. Prepulse Inhibition Procedure

Ventilated and lighted startle chambers (SR-LAB; San Diego Instruments, San Diego, Calif, USA) were utilized for all PPI experiments. Each chamber (33 × 33 × 46 cm) was equipped with a loudspeaker (acoustic source) and a Plexiglas cylindrical animal enclosure (internal diameter: 8.8 cm for rat, 3.8 cm for mouse) mounted on a Plexiglas base. Startle responses were transduced by a piezoelectric accelerometer mounted below the cylinder. The loudspeaker was positioned above the cylinder and produced the mixed frequency stimuli (background noise, prepulse and pulse stimuli).

Test sessions began with a 5 min acclimation period, during which a background noise was presented in the absence of any startle stimuli. The animals were then subjected to a series of acoustic startle trials. For mouse PPI, the animals received six trial types: no stimulus, startle alone (130 dB, 40 ms), highest prepulse alone (20 ms), and three different prepulses (5 dB, 10 dB, and 15 dB, 20 ms) preceding a startle stimulus by 100 ms. Each trial type was presented in a pseudorandom order with 12 presentations of each, in addition to an initial single pulse alone trial which began the test session. This initial pulse trial was not used in data analysis. The intertrial interval averaged 18 s (10–25 s range). For rat PPI, a total of 41 trials were presented. They consisted of five trial types: no stimulus, startle alone (120 dB, 40 ms), and 3 prepulse stimuli (5, 10, and 15 dB above 65 dB background, 20 ms), each preceding the startle stimulus by 100 ms. Each trial type was presented in a pseudorandom order with 8 presentations of each in addition to an initial single pulse alone trial, which was not used for data analysis. The average intertrial interval was 20 s (15–25 s range). PPI data are expressed as an average of the percent inhibition of startle produced by the 5, 10, and 15 dB prepulse trials. Mean startle magnitude was calculated based on the startle alone trials. All animals were initially subjected to a baseline testing day without pharmacological manipulation in order to create groups with equivalent mean baseline levels of startle and PPI. All studies were conducted using a between-subjects design.

### 2.5. Statistical Analysis

All data are expressed as means ± the standard error of the mean (SEM). All studies were analyzed using one-way ANOVAs. Dunnett's tests were used to determine individual dose groups with significant reductions in time cataleptic compared to the haloperidol + vehicle group for catalepsy studies or individual dose groups with significant reductions in percent PPI or startle compared to the vehicle group in PPI studies. Significance was defined as *P* < 0.05. 

## 3. Results

### 3.1. Haloperidol-Induced Catalepsy

**Figure 1:**
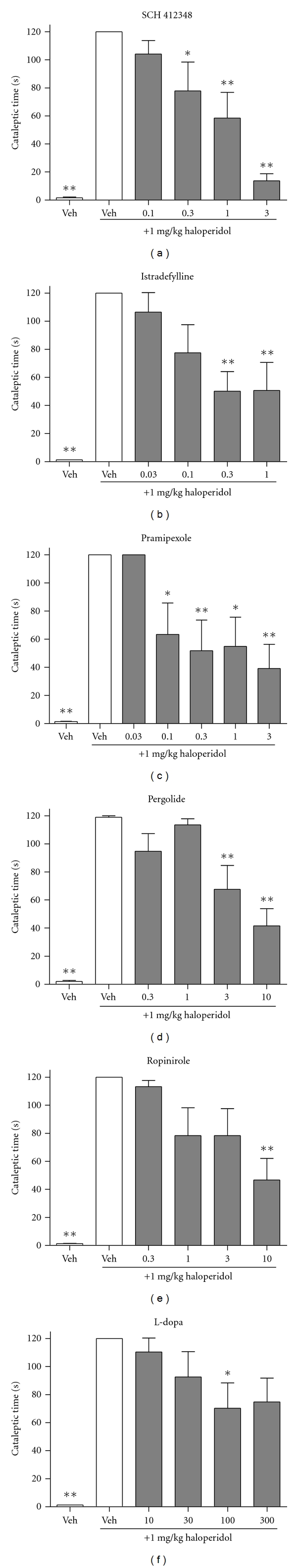
Efficacy of SCH 412348 (a), istradefylline (b), pramipexole (c), pergolide (d), ropinirole (e), and L-dopa (f) to reduce catalepsy induced with 1 mg/kg haloperidol in rats. Data represent mean time cataleptic ± SEM and were analyzed by one-way ANOVAs with Dunnett's tests (*P < 0.05; **P < 0.01 versus vehicle + haloperidol treatment).

Figure 1 shows treatment effects on rat haloperidol-induced catalepsy. The A_2A_ antagonists SCH 412348 (Figure 1(a)) and istradefylline (Figure 1(b)) significantly reversed rat haloperidol-induced catalepsy (SCH 412348: *F*(5, 42) = 15.57, *P* < 0.01; istradefylline: *F*(5, 42) = 9.20, *P* < 0.01), with the 0.3, 1, and 3 mg/kg groups and the 0.3 and 1 mg/kg groups, respectively, spending significantly less time cataleptic than the vehicle + haloperidol group. The DA receptor agonists pramipexole, pergolide and ropinirole also reduced haloperidol-induced catalepsy in rats. Pramipexole effects (*F*(6, 35) = 7.57, *P* < 0.01) were significantly different from vehicle at 0.1, 0.3, 1, and 3 mg/kg (Figure 1(c)), while pergolide (*F*(5, 42) = 19.98, *P* < 0.01) showed significant effects compared to vehicle at doses of 3 and 10 mg/kg (Figure 1(d)). Ropinirole reduced time cataleptic (*F*(5, 30) = 11.42, *P* < 0.01) at 10 mg/kg, whereas 1 and 3 mg/kg showed marginal significance (*P* = 0.06) compared to vehicle + haloperidol treatment (Figure 1(e)). L-dopa significantly reduced haloperidol-induced catalepsy (*F*(5, 50) = 7.13, *P* < 0.01) at 100 mg/kg, whereas 300 mg/kg approached significance (*P* = 0.06) compared to vehicle + haloperidol treatment (Figure 1(f)).

### 3.2. Rat PPI

**Figure 2:**
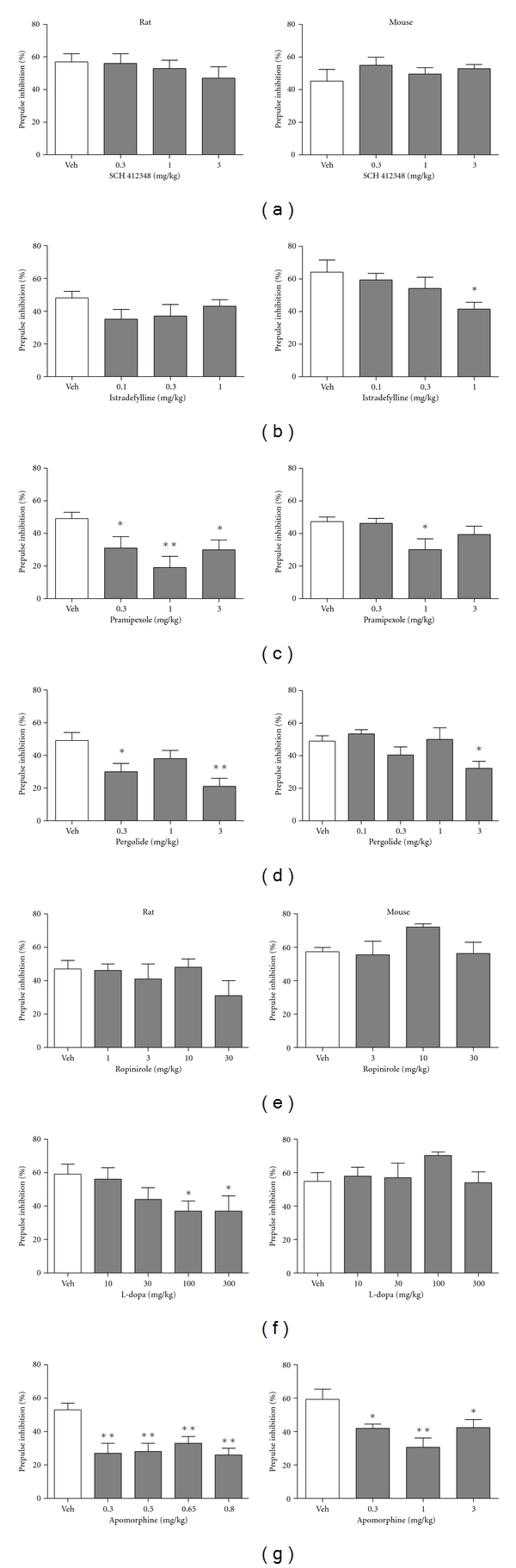
Effects of SCH 412348 (a), istradefylline (b), pramipexole (c), pergolide (d), ropinirole (e), L-dopa (f), and apomorphine (g) on rat (left) or mouse (right) prepulse inhibition. Data represent mean % PPI ± SEM and were analyzed by one-way ANOVAs with Dunnett's tests (*P < 0.05; **P < 0.01 versus vehicle treatment).

Figure 2 (left graphs) shows treatment effects on rat PPI, and Table 1 shows treatment effects on rat startle magnitude. The A_2A_ antagonists SCH 412348 (Figure 2(a)) and istradefylline (Figure 2(b)) did not impair rat PPI (SCH 412348: *F*(3, 28) = 0.57, *P* > 0.05; istradefylline: *F*(3, 28) = 1.18, *P* > 0.05) or startle magnitude (SCH 412348: *F*(3, 28) = 0.31, *P* > 0.05; istradefylline: *F*(3, 28) = 0.20, *P* > 0.05) at any doses tested. Pramipexole (Figure 2(c)) significantly reduced PPI at all doses tested (0.3, 1, and 3 mg/kg) (*F*(3, 60) = 4.47, *P* < 0.01) but also significantly reduced startle magnitude at all doses tested (*F*(3, 60) = 5.24, *P* < 0.01) compared to vehicle, which is consistent with previous findings [11]. Pergolide (Figure 2(d)) impaired PPI at 0.3 and 3 mg/kg (*F*(3, 60) = 5.64, *P* < 0.01) but did not affect startle (*F*(3, 60) = 1.29, *P* > 0.05). Ropinirole (Figure 2(e)) did not impair PPI in rats (*F*(4, 32) = 1.16, *P* > 0.05) but reduced startle magnitude at 3 and 30 mg/kg (*F*(4, 32) = 4.61, *P* < 0.01). For L-dopa (Figure 2(f)), a one-way ANOVA revealed only a marginally significant effect on PPI overall (*F*(4, 35) = 2.46, *P* = 0.06). The two highest doses of L-dopa (100 and 300 mg/kg) significantly disrupted PPI. Startle was not affected by treatment with L-dopa (*F*(4, 35) = 0.23, *P* > 0.05). Apomorphine (Figure 2(g)) significantly reduced PPI in rats (*F*(4, 75) = 5.58, *P* < 0.01) at all doses tested (0.3, 0.5, 0.65, and 0.8 mg/kg) but had no effect on startle (*F*(4, 75) = 0.76, *P* > 0.05).

### 3.3. Mouse PPI

Figure 2 (right graphs) shows treatment effects on mouse PPI, and Table 2 shows treatment effects on mouse startle magnitude. SCH 412348 (Figure 2(a)) did not significantly decrease PPI or startle in mice (PPI: *F*(3, 36) = 0.74, *P* > 0.05; startle: *F*(3, 36) = 0.14, *P* > 0.05). Istradefylline (Figure 2(b)) approached overall significance in reducing mouse PPI (*F*(3, 36) = 2.83, *P* = 0.05). Istradefylline (1 mg/kg) significantly reduced PPI compared to vehicle. Startle was not affected by istradefylline at any dose (*F*(3, 36) = 1.26, *P* > 0.05). Pramipexole (Figure 2(c)) significantly reduced PPI in mice only at 1.0 mg/kg (*F*(3, 44) = 2.91, *P* < 0.05). Unlike its effects on rat startle magnitude, pramipexole did not significantly reduce mouse startle (*F*(3, 44) = 2.53, *P* > 0.05). Pergolide (Figure 2(d)) significantly reduced mouse PPI and startle at 3 mg/kg (PPI: *F*(4, 43) = 3.40, *P* < 0.05; startle: *F*(4, 43) = 3.63, *P* < 0.05). Ropinirole (Figure 2(e)) did not significantly reduce mouse PPI (*F*(3, 36) = 2.09, *P* > 0.05) or startle magnitude (*F*(3, 36) = 0.05, *P* > 0.05) at any dose tested. For L-dopa (Figure 2(f)), there was no significant main effect of dose on PPI (*F*(4, 42) = 1.01, *P* > 0.05). However, startle was significantly reduced by 100 and 300 mg/kg of L-dopa (*F*(4, 42) = 5.48, *P* < 0.01). Apomorphine (Figure 2(g)) significantly reduced mouse PPI at all doses tested (0.3, 1, and 3 mg/kg) (*F*(3, 43) = 5.96, *P* < 0.01) but did not affect startle magnitude (*F*(3, 43) = 2.58, *P* > 0.05).

## 4. Discussion

A_2A_ receptor antagonism has received considerable recent attention as an alternative treatment for the motor symptoms of PD [18, 19]. A_2A_ receptor antagonists have proven to be efficacious in animal models of PD and in clinical studies. Because they represent a nondopaminergic approach to the treatment of PD, we hypothesized that A_2A_ receptor antagonists will avoid neuropsychiatric side effects associated with dopaminergic therapies, including psychosis. The findings of the present studies are consistent with this hypotheisis.

PPI can be measured in humans, rats, mice, and other mammals and is deficient in pathological or drug-induced psychotic states. In the current studies, pramipexole, pergolide, and apomorphine disrupted PPI in both rat and mouse. These results are consistent with previous findings in rats [10, 11, 20]. Moreover, the disruptive effects occurred at doses that were efficacious in rat haloperidol-induced catalepsy, a rodent model of PD. Although Swerdlow et al. [10] found that ropinirole (3–6 mg/kg) induced deficits in rat PPI, ropinirole did not impair PPI in the present studies, even when tested up to 30 mg/kg in the rat and mouse. The different effects on PPI between ropinirole and other DA receptor agonists are consistent with their clinical profiles. PD patients treated with pramipexole have a significantly higher risk of experiencing hallucinations than patients treated with ropinirole [21]. In addition, ropinirole is less likely to induce psychosis when used as monotherapy for PD than when administered adjunctively with other DA receptor agonists [22]. Like pramipexole and pergolide, ropinirole is a potent D_2_ and D_3_ receptor agonist that favors the D_3_ receptor, as does the major metabolite of ropinirole [23]. As such, the difference between ropinirole's psychosis liability as compared to other DA receptor agonists is not clearly understood.

We found that L-dopa produced a marginal disruption of rat PPI but did not disrupt mouse PPI. There are conflicting reports about the relative psychosis liability of L-dopa versus DA receptor agonists. Some findings indicate that L-dopa has similar potential to DA receptor agonists to elicit psychosis [24], whereas other studies suggest that DA agonists are more likely to induce psychosis than L-dopa [5–7].

The clear lack of PPI disruption with SCH 412348 is interesting considering that the efficacy of both DA receptor agonists and A_2A_ antagonists is hypothesized to be mediated by similar effects at the second messenger level. The receptors are colocalized on neurons in the striatopallidal indirect pathway of the basal ganglia. A_2A_ receptor antagonists and D_2_ receptor agonists decrease intracellular adenylyl cyclase activation [25]. These pharmacological approaches evoke similar behavioral profiles in rodents and primates. Several findings provide evidence for extrastriatal DA receptor involvement in PPI [26]. It is possible, therefore, that neuropsychiatric side effects associated with DA receptor agonists are, at least partially, mediated by activity outside the striatum. Unlike D_2_ receptors, A_2A_ receptors are predominantly localized in the striatum [14], which could explain their benign neuropsychiatric side effect profile.

The distinction between the two approaches may be attributed to different effects on the various DA receptor subtypes. While it is well established that there is a functional A_2A_-D_2_ receptor interaction, the relationship between A_2A_ and D_3_ receptors is less well understood [27]. Chang et al. [20] demonstrated that PPI using acoustic startle is highly sensitive to activation of the D_3_ receptor. More research is necessary to better understand the pharmacology responsible for the PPI-disruptive effects of agonism at various DA receptor subtypes as well as the difference between selective A_2A_ antagonists and DA receptor agonists reported herein.

Interestingly, istradefylline induced a marginal disruption of mouse PPI at the highest dose tested (1 mg/kg). The dissimilar effects of SCH 412348 and istradefylline in rodent PPI may be due to their relative activity at the adenosine A_1_ receptor. Whereas SCH 412348 is greater than 1000-fold selective for the A_2A_ receptor over the A_1_ receptor, istradefylline exhibits only 82-fold selectivity [17]. Koch and Hauber [28] found that the nonselective adenosine receptor antagonist, theophylline, potentiated an apomorphine disruption of PPI. This effect was reversed by a selective A_1_ receptor agonist but not a selective A_2A_ receptor agonist. Collectively, these data suggest that istradefylline's activity at the A_1_ receptor may have contributed to the disruption of PPI observed at the highest dose tested.

Although psychotic symptoms may also occur in PD patients in the absence of pharmacological treatment, it is still uncertain if the pathology of the disease itself predisposes the patients to developing neuropsychiatric symptoms with dopaminergic treatment. Studies have suggested that PD-associated psychosis results from interactions between pharmacological and disease-related factors [29]. Considering that the present studies were performed using healthy animals, future PPI studies using an animal model of PD, such as the MitoPark mouse, which has a gradual degeneration of dopamine cells and a parkinsonian phenotype [30], may help elucidate the contribution of the disease to the neuropsychiatric effects of dopaminergic treatment. Marcellino et al. [31] reported that chronic treatment with an A_2A_ antagonist alleviated the motor deficits of MitoPark mice. Therefore, a comparison of the effects of A_2A_ receptor antagonists to dopamine receptor agonists in the sensory gating PPI model using MitoPark mice would be beneficial in further understanding the potential benefits of A_2A_ receptor antagonism as a treatment for PD without increased risk of developing psychosis.

## 5. Conclusions

The highly selective A_2A_ receptor antagonist, SCH 412348, did not induce a PPI deficit in either the rat or mouse. Conversely, DA receptor agonists used for the treatment of PD demonstrated disruptive effects in PPI. Istradefylline modestly disrupted rodent PPI, which we attribute to its activity at the adenosine A_1_ receptor. Clearly, more work is required to understand the pharmacology of the disruptive effects of different antiparkinsonian agents. Collectively, our data indicate that A_2A_ receptor antagonism is a promising nondopaminergic treatment for PD that may avoid neuropsychiatric side effects provided that the antagonist has sufficient selectivity over the A_1_ receptor.

## Figures and Tables

**Table 1:** Mean startle magnitude ± SEM in rat prepulse inhibition (*P < 0.05 versus vehicle).

Drug	Dose (mg/kg)	Startle
SCH 412348	Veh.	193.3 ± 46.6
	0.3	223.4 ± 59.5
	1.0	166.0 ± 20.2
	3.0	213.0 ± 46.3

Istradefylline	Veh.	196.5 ± 50.8
	0.1	163.7 ± 26.7
	0.3	161.5 ± 23.3
	1.0	175.0 ± 36.9

Pramipexole	Veh.	229.5 ± 32.1
	0.3	121.4 ± 14.4*
	1.0	134.7 ± 32.0*
	3.0	101.6 ± 14.2*

Pergolide	Veh.	139.9 ± 20.7
	0.3	104.9 ± 27.1
	1.0	111.0 ± 19.1
	3.0	84.0 ± 11.1

Ropinirole	Veh.	250.2 ± 49.7
	1.0	218.8 ± 33.7
	3.0	73.0 ± 17.6*
	10.0	166.6 ± 26.7
	30.0	111.4 ± 22.1*

L-dopa	Veh.	228.7 ± 124.7
	10	183.5 ± 31.0
	30	269.8 ± 63.7
	100	239.0 ± 45.1
	300	197.2 ± 49.8

Apomorphine	Veh.	303.2 ± 46.3
	0.3	323.5 ± 44.3
	0.5	456.3 ± 140.0
	0.65	468.1 ± 76.2
	0.8	355.2 ± 79.6

**Table 2:** Mean startle magnitude ± SEM in mouse prepulse inhibition (*P < 0.05 versus vehicle).

Drug	Dose (mg/kg)	Startle
SCH 412348	Veh.	129.0 ± 17.8
	0.3	144.6 ± 17.8
	1.0	140.5 ± 20.7
	3.0	142.6 ± 12.9

Istradefylline	Veh.	125.9 ± 22.3
	0.1	104.9 ± 17.3
	0.3	110.9 ± 19.0
	1.0	75.9 ± 10.7

Pramipexole	Veh.	95.6 ± 8.7
	0.3	103.1 ± 13.2
	1.0	76.3 ± 8.5
	3.0	69.5 ± 10.5

Pergolide	Veh.	135.2 ± 23.5
	0.1	149.9 ± 12.3
	0.3	116.9 ± 17.6
	1.0	100.5 ± 15.1
	3.0	62.7 ± 10.9*

Ropinirole	Veh.	135.9 ± 19.7
	3.0	134.1 ± 15.2
	10.0	143.3 ± 15.6
	30.0	140.6 ± 18.0

L-dopa	Veh.	130.5 ± 20.9
	10.0	135.0 ± 16.4
	30.0	96.8 ± 14.4
	100.0	69.0 ± 5.8*
	300.0	50.7 ± 9.6*

Apomorphine	Veh.	149.9 ± 26.4
	0.3	93.1 ± 15.9
	1.0	90.6 ± 15.2
	3.0	94.7 ± 13.5
